# Ever-present threats from information technology: the Cyber-Paranoia and Fear Scale

**DOI:** 10.3389/fpsyg.2014.01298

**Published:** 2014-11-24

**Authors:** Oliver J. Mason, Caroline Stevenson, Fleur Freedman

**Affiliations:** Department of Clinical, Educational and Health Psychology, University College LondonLondon, UK

**Keywords:** paranoia, information technology, psychometrics, internet, self-reported symptom

## Abstract

Delusions involving technology, and specifically the internet, are increasingly common, and fear-reality statistics suggest computer-related fears are very widespread. These fears form a continuum from the widely understandable and realistic to the unrealistic, and frankly paranoid. The present study investigated the validity of this construct in a non-clinical population by constructing a novel self-report measure. The new Cyber-Paranoia and Fear Scale aims to measure the perception of information technology-related threats originating from or enabled by computers, smartphones, social networks, and digital surveillance. Psychometric properties of the new Cyber-Paranoia and Fear Scale are reported alongside an established measure of suspiciousness and paranoia in 181 participants including a sub-group of fifty information technology professionals. Exploratory factor analysis suggested the presence of two, related, dimensions that we term cyber-paranoia and cyber-fear. Both sub-scales were internally consistent and produced a normal distribution of scores. The relationships of the sub-scales with age, gender, trait paranoia, digital literacy, and digital inclusion are supportive of construct validity. The distinctiveness of ‘cyber-paranoia’ from general trait paranoia appears to mirror the clinical distinctiveness of ‘internet’ and other technology-fuelled delusions. Knowledge provision to increase technological proficiency and awareness may bring about a reduction in cyber-paranoia.

## INTRODUCTION

The exponential development of technology has seen numerous reports of its incorporation into clinical paranoia and delusional thinking. Some social science commentators have also suggested an excessive level of fear regarding modern technology and cyber-crime within the general population. Stewart and Segars (2002) term this *computer anxiety*, and suggest that this can influence intentions to use cyber-technology. Related to this are individuals’ concerns about their privacy online with several attempts to measure this (Smith et al., 1996; Stewart and Segars, 2002). The earliest privacy theorist, Westin (2003), described the most protective of their privacy and distrustful of organizations as ‘privacy fundamentalists.’ Smith et al. (1996) found concerns about privacy to stem from traits of distrust, paranoia, and social criticism. We have aimed assess cyber-related feelings, attitudes, beliefs, and behaviors that stem particularly from distrust, fear, and paranoia. We have termed the extreme of these *cyber*-*paranoia* (named after the quasi-clinical results that may ensue when fears go unchecked). By cyber-paranoia we mean unrealistic fears concerning threats via information technologies whereby individuals perceive themselves to be open to be ‘attacked,’ persecuted or victimized in some way. However, the boundaries of what is a realistic fear are increasingly blurred with an accurate perception of risk probably only afforded to those in information technology security. Ultimately the estimation of what is realistic or not by way of threat is to some degree at least subjective (just as ultimately all paranoid beliefs are), and as a consequence we have not *a priori* set out to define these rigidly. Properly then, we aim to capture a range of fears of varying in subjective realism and likelihood. We aim to measure and validate this new measure – the Cyber-Paranoia and Fear Scale – alongside the widely accepted trait construct of general paranoia, and a range of indices of digital literacy and inclusion. What we are *not* advancing is that cyber-paranoia has necessarily a wholly different etiology or psychology to trait paranoia, rather that the phenomenon may be sufficiently different in content and form to warrant specific measurement and thus further study. By way of background we outline some thoughts on the parasocial nature of cyberspace; some clinical observations concerning fears of technology, and computers in particular; followed by a discussion of paranoia in the general population.

### PROBLEMATIC RELATING IN CYBER-SPACE

While existing relationships to individuals and organizations may extent into cyber-space, it also provides limitless opportunities for contact, even attachments to form, largely or solely online. The range of social and parasocial relationships probably engender the full gamut of emotional (Benski and Fisher, 2013) and behavioral responses including dependency (Reynolds et al., 2007), fear and paranoia (Berner, 2009). Perhaps the relative novelty of the internet helps explain the widespread lack of clarity about the nature of these relationships as well as issues like reputation, trust, privacy, and responsibility. Such issues form a particular focus on social media sites (e.g., Facebook and MySpace Dwyer et al., 2007). A recent study (Martin et al., 2012) of social networking profiles found that paranoia and suspiciousness predicted the number of Facebook lines ‘blacked out’ using privacy settings. Recent cases of persecution using Twitter have led to questions about ethics, responsibilities, and legalities online. A White House national security official was fired after it was discovered that he was behind an anonymous Twitter account that criticized the Obama administration (e.g., http://www.bbc.co.uk/news/world-us-canada-24637160). Probably the most widespread problematic behaviors on the part of users to date (at least in what is researched here) are cyber addictions (Lortie and Guitton, 2013; gambling, gaming, cyber-sex, and generally excessive internet or even smartphone usage Billieux, 2012) and bullying. Our main point here is that a wide range of problematic behaviors (perhaps most that occur in the non-cyber world) characterize cyber-space with these exploratory studies suggesting concerns of persecution/paranoia are very widespread.

### TECHNOLOGY IN PARANOID DELUSIONS

As the use of micro-chip and internet technology has become increasingly more pervasive in society, the literature has seen an increasing number of reports of paranoid delusions with technology as a central theme (Catalano et al., 1999; Compton, 2003; Lerner et al., 2006; Nitzan et al., 2011). The commonest theme is of being controlled by the internet. Ideas of reference and control permeate these accounts which frequently extend beyond the internet to involve electronics/micro-chips and other persecutory agents using internet-based forms of surveillance and control. Lerner et al. (2006) predicted that developments in the use of technology in our daily lives would, in turn, see developments in the incorporation of technology into delusions. This prediction is supported by recent studies suggesting increasing reference to social networking media. For example, Nitzan et al. (2011) described three such cases characterized by ‘hyper-personal’ relationships with strangers and blurred self-boundaries with regards to social networking media.

What almost all reported cases have in common is a relative lack of familiarity with technology and with the internet. Indeed, both Catalano et al. (1999) and Compton (2003) postulated that this lack of knowledge may fuel internet-themed delusions. Nitzan et al. (2011) also suggested the role of technical difficulties, and specifically difficulties in deciphering the meaning of various elements of social networking, in increasing patients’ vulnerability. However, delusions regarding technology (and specifically the internet) are relatively modern phenomena, and there is no consensus on their status. Compton (2003) regarded the internet as simply the socio-cultural content of otherwise familiar paranoid delusions, and Lerner et al. (2006), Nitzan et al. (2011, p. 1) agreed, arguing that internet delusions are just “modified delusions of persecution, broadcasting, and control.” However, Catalano et al. (1999) alluded to internet delusions as a subtype of delusional thinking, echoing a similar idea to internet addiction being a novel form of addiction, due to the unique features of the internet which may lead people to use it compulsively in order to feed a social or emotional need (Shapira et al., 2003). Furthermore, Duggal et al. (2002) suggested that the presence of internet-themed delusions may be a specific prognostic indicator, noting particular success using cognitive therapy as a treatment. The authors classified this as ‘perception broadcast,’ a term which they coined after noting the involvement of perceptions rather than thoughts, and the lack of direct participation of others. Based on a case series of ‘internet’ delusions, Bell et al. (2005) also felt that their form, origin and content were influenced by the technology involved, and thus well suited to psycho-educational treatment.

### PARANOIA IN THE GENERAL POPULATION

Interview and questionnaire research has typically reported regular paranoid thinking in around 15–20% of the general population (Freeman, 2007). This degree of prevalence has led many researchers to propose that paranoia is a personality trait found on a continuum throughout the population to varying degrees. For example, Freeman et al. (2005) found that 30–40% of a student sample experienced social evaluative concerns, such as ideas of social reference, 10–30% experienced mild persecutory delusions and 5% experienced strong persecutory thoughts weekly. Using the Adult Psychiatric Morbidity Survey in England, Freeman et al. (2011) reported prevalence of paranoid thinking in the previous year to range from 18.6% (people were against me), to 1.8% (potential plots to cause me serious harm). The nature of the hierarchy of paranoid concerns, and what constitutes the essential components of trait paranoia/paranoid thinking in the general population has been increasingly researched and discussed in recent years. This literature has turned its attention to a range of psychological factors implicated in paranoid thinking: adverse early experiences such as abuse, victimization and bullying; emotional processes such as interpersonal sensitivity, anxiety and depression; negative beliefs about the self and others; and biases in reasoning [see Freeman (2007) for review]. Interestingly computer-based virtual reality scenarios have increasingly been used to generate and study paranoid thinking, originating with a study Freeman et al. (2003). An increasing range of measures capturing a spectrum of potentially paranoid concerns have been used in the literature: one early and commonly used measure, the Paranoia Scale (Fenigstein and Vanable, 1992) indexes socially evaluative beliefs and experiences that range from mildly anxious to persecutory in nature. The Paranoia Scale is based on the theory that there is a continuum of severity across a wide range of concerns, and we retained this broadly phenomenological approach to the development of our own scale.

### TECHNOLOGICAL FEARS – JUSTIFIED OR PARANOID?

Studies of paranoia in the non-clinical population have generally focused on unrealistic fear appraisals. Successful adoption of technology requires an attitude of confidence and the expectation that one’s vulnerabilities in an online situation will not be exploited (Horn, 1965): however, there seems to be widespread lack of trust which is out of proportion to the actual risks. Based on the findings of a national cyber-crime victimization survey in 2004 from a national list of people who reported having internet access Alshalan (Velicer, 1976) found that both older people and women exhibited the greatest fear; despite young males being the most often victimized. Most available evidence suggests that cyber-crime is actually statistically rather rare: In 2003, The United States Federal Trade Commission found that less than 1% of reported cases of identity fraud could be linked to the internet but that risk is over-estimated (O’Connor, 2000). In fact, only 8% of identity theft victims surveyed by Lewis and Fox (2001) and Corritore et al. (2003) had evidence that even indicated the internet *might* have been involved. However, it is genuinely difficult to assess what is possible technologically and how frequently it may occur. Is it any surprise then that technological advancement, which arguably should generate a feeling of empowerment, perhaps generates, at least for some a profound and general sense of powerlessness and vulnerability (Alshalan, 2006)?

When asked about the perceived risk and ‘seriousness,’ nearly three quarters of one US survey (Velicer, 1976) perceived most computer crimes as serious compared to equivalent ‘street’ crimes. Ohm’s review (Roberts et al., 2013) pointed out many ‘online’ harms are ones of an emotionally charged nature, and are likely to bypass realistic assessment of probabilities. In a wide review of attitudes to global information technology, Lewis and Fox (2001) and Taipale (2005) came to these conclusions in regards to technology and the general public: “The availability of information privacy horror stories […], and the general mistrust in government agencies to handle personal information appropriately, combined with a general apprehension about technology and how it works, and the natural anxiety relating to disclosure of personal, particularly intimate, information […] has created a public anxiety about electronic privacy out of proportion to the actual risks” (p. 137).

On balance, the weight of the evidence points to an excessive level of fear regarding information technology within society, in that the level of fear seems to be out of proportion to the actual risks. We aimed to specify and quantify these relatively common fears so as to develop a novel measure of cyber-paranoia; in particular addressing the nature of its relationship to trait paranoia and use of information technology more generally.

## MATERIALS AND METHODS

Data was collected both using an anonymous internet survey and via snowballing to aid recruitment, in particular by attracting information technology professionals. Additionally, the survey was promoted using social-networking media, advertised by posters (in University computer rooms) and it was also spread via word of mouth. All promotional attempts were based in the UK, though it is possible international respondents became aware of the online study. The IT professionals were recruited via an online social network of employees of a number of IT companies in the UK. No participants were paid for their participation. The study was approved within the Division of Psychology and Language Sciences, University College London.

### PARTICIPANTS

One hundred and eighty one (75 females, 106 males) respondents aged between 18 and 83 (mean age = 30.2, SD = 14.3) completed the online survey. Fifty participants self-identified as IT professionals.

### MEASURES

#### Cyber-Paranoia and Fear Scale

This self-report measure was devised specifically for the present study. It investigates the prevalence of paranoid beliefs pertaining to relatively modern forms of communication, information, and surveillance. An item pool of 26 items was generated in consultation with social scientists interested in information technology, and by consulting with technology users about common fears. Items were rated using a four-point Likert-style scale (“Strongly Disagree,” “Slightly Disagree,” “Slightly Agree,” “Strongly Agree”). A four-point scale was used as this omits a neutral point and thereby reduces any tendency to a non-committal stance on a subject. Exploratory Factor analysis (EFA) was used to select from this pool, items that had strong unique factor loadings (main loading < 0.4, secondary loading < 0.02) and factorially relevant and coherent content so as to form scales with sufficient discriminant validity. This resulted in the selection of six items to measure cyber-paranoia, and five items to measure cyber-fear.

#### General trait paranoia

The 20 item Paranoia scale (Fenigstein and Vanable, 1992) was developed to measure paranoia in college students, and includes items assessing both ideas of persecution and reference. The Paranoia Scale is the most widely used dimensional measure of paranoia and was derived from items in the Minnesota Multiphasic Personality Inventory (MMPI). Fenigstein and Vanable (1992) report good internal consistency (based on a total student sample of 581) and a Cronbach’s alpha of 0.84 and test- re-test reliability (based on a 6-month re-test, *n* = 107) of 0.70, indicating good stability over time.

Awareness of technology, years of internet use and frequency of internet use were assessed by single item five-point Likert scales.

## RESULTS

Consistent with a technologically based recruitment strategy primarily located at a university, the majority of the participants were in higher education (81%) and considered their awareness of technology as above average (62%), with 29% describing it as ‘very high.’ 81% of participants reported internet use for at least 7 years, and 76% reported using it for several hours per day. In addition, 77% of the sample reported owning a smartphone. Descriptives, including the subscales derived from factor analysis, are given in **Table 1**. The means and standard deviations seen for the Paranoia Scale are similar to those previously reported in the general population (Fenigstein and Vanable, 1992).

**Table 1:** Descriptives.

Scale (range)	General population (*n* = 131)	IT professionals (*n* = 50)
Age	29.2 (14.7)	32.7 (12.9)
Cyber-Paranoia Subscale (6–24)	13.3 (3.7)	12.5 (3.7)
Cyber-Fear Subscale (5–20)	13.4 (3.2)	14.7 (2.7)
Paranoia Scale* (20–100)	41.7 (14.6)	38.2 (13.1)
Awareness of technology (1–5)	3.8 (0.93)	4.6 (0.68)

*Fenigstein and Vanable (1992).

The newly devised items were submitted to EFA. Both Horn’s Parallel Analysis (Horn, 1965) and Velicer’s minimum average partial (MAP) test (Velicer, 1976) were conducted using O’Connor’s (2000) SPSS program to identify the number of factors with eigenvalues significantly above chance levels. Parallel analysis, Velicer’s original MAP test and O’Connor’s revised MAP test all supported the extraction of two factors. Subsequent EFA was conducted both using principal axis analysis and Promax rotation, and, as a further check with maximum likelihood analysis with Varimax rotation which produced highly similar results. Inspection of the items led to the factors’ description as *cyber-paranoia* and *cyber-fear*: subscales for these were constructed by selecting items with coherent and relevant content and that had strong and unique factor loadings. Items were rejected where either the main loading was insufficient (<0.4) or there was evidence of mixed loading (secondary loading > 0.2).

This process led to the final set of selected items shown in **Table 2**. EFA of these items explained 36.0% of the variance, with the pattern matrix factor loadings shown in **Table 2**. The factors are significantly inter-correlated (*r* = 0.45). This led to subscales formed of six cyber-paranoia and five cyber-fear items: both have adequate internal consistency (Alpha coefficients of 0.75 for cyber- paranoia and 0.74 for Cyber- Fear). Levels of skewness and kurtosis were also highly acceptable (<±1).

**Table 2:** Pattern matrix factor loadings.

Items	Factor	
	1	2
Increasing computer usage is changing children’s brains for the worse	**0.68**	–0.08
It’s only a matter of time until the global web is brought down with dire consequences	**0.64**	0.00
I avoid using the internet on personal matters so as not to have my details accessed	**0.58**	–0.05
I worry about others editing my Facebook page (or similar) without my consent	**0.55**	0.03
I worry about the effects of electromagnetic waves from mobile phones/phone masts	**0.53**	–0.04
Terrorists will find new ways to use the internet to plan new attacks on the general public	**0.48**	0.15
		
Payment cards such as Oyster cards allow the authorities to monitor my travel and purchases	–0.15	**0.71**
Companies that store data on customers are very vulnerable to theft of my private details	0.08	**0.62**
People do not worry enough about threats from their use of technology	0.19	**0.53**
People should worry that their movements can be monitored via their ‘smartphone’	–0.07	**0.59**
Closed circuit television cameras (CCTV) are illegally used to spy on people	0.01	**0.57**

Differences on the subscales were investigated with respect to the two groups of participants (general population vs. IT professionals) and gender using ANOVA. For the Cyber-Paranoia subscale there was a main effect of gender (*F* = 7.2, *p* = 0.008), with higher scores in females than males (14.3 vs. 12.4). However, for the Cyber-Fear scale, there was a significant main effect of group (*F* = 6.0, *p* = 0.015), with IT professionals slightly exceeding the general population sample substantiating that these are realistic fears held by knowledgeable respondents. The concurrent validity of subscales was investigated by comparing its subscales with the Paranoia Scale (see **Table 3**). In the general population these relationships were very modest, only reaching significance for cyber-fear (*r* = 0.2). Interestingly cyber-paranoia correlated moderately with general trait paranoia in the group of IT professionals (*r* = 0.59). While older participants in both groups were somewhat less paranoid in general, this did not extend to cyber-fear and cyber-paranoia, indeed in the general population sample cyber-paranoia increased with age. In addition, the Cyber-Fear and Cyber-Paranoia subscales produced quite different patterns of relationships with technology awareness and internet use to general trait paranoia that were also group dependent (see **Table 3**). In the general population cyber-fear was associated with fewer years of internet use, and cyber-paranoia with less awareness, fewer years and lower frequency of internet use. These findings were not seen in the IT professionals for whom many of these indices were understandably somewhat at ceiling. Smartphone users were significantly less cyber-fearful and cyber-paranoid than non-users [*t*(129) = 2.60, *p* = 0.01; *t*(129) = 2.83, *p* = 0.005] – a difference not seen for the Paranoia Scale [*t*(129) = 0.358, *p* = 0.721].

**Table 3:** Correlations in general population and IT professionals.

General population (*n* = 131)	Age	Cyber-paranoia	Cyber-fear	Paranoia Scale
Cyber-paranoia	0.38**	–	0.37**	0.01
Cyber-fear	0.11	0.37**	–	0.20*
Paranoia Scale	–0.34**	0.01	0.20*	–
Technology awareness	–0.24**	–0.34**	–0.02	0.03
Years of internet use	–0.29**	–0.41**	–0.25**	0.02
Frequency of internet use	–0.61**	–0.35**	–0.11	0.22*
**IT professionals (*n* = 50)**				
Cyber-paranoia	–0.22	–	0.40**	0.59**
Cyber-fear	0.03	0.40**	–	0.23
Paranoia Scale	–0.39**	0.59**	0.23	–
Technology awareness	–0.17	–0.11	0.25	0.05
Years of internet use	0.10	–0.19	–0.01	0.03
Frequency of internet use	–0.57**	0.04	0.06	0.17

*p<0.05, **p<0.01.

## DISCUSSION

There is much clinical evidence of technology being incorporated into paranoid delusions at the more severe end of psychosis (Catalano et al., 1999; Compton, 2003; Lerner et al., 2006; Nitzan et al., 2011), and fear-reality statistics indicate a general apprehension with regards to technology that is out of proportion to the actual risks (Velicer, 1976; O’Connor, 2000; Corritore et al., 2003). However, the present study is the first to investigate this observed ‘cyber-paranoia’ phenomenon quantitatively. In addition to establishing whether these fears could be measured reliably, the study aimed to address whether ‘cyber-paranoia’ is simply another aspect of non-clinical paranoia or is, to some degree at least, a separable construct. On the present evidence, the relationship is relatively modest at least in the general population and suggests that much variance may be relatively unique to cyber-fear and cyber-paranoia. Intriguingly there was a much greater relationship with general trait paranoia in IT professionals for whom technological awareness is uniformly greater. This may suggest that when IT knowledge is uniformly very high, as it is for the IT professionals, their non-rational paranoia perceptions result from their general trait paranoia. Moreover the Cyber-Paranoia Scale produced a different pattern of relationships with age and technological use and awareness. Unlike general trait paranoia, in the general population cyber-fear and cyber-paranoia were associated with lower internet and smartphone use as well as lower familiarity. This perhaps mirrors the findings of Freeman et al. (2003), whereby participants with less experience of using underground trains displayed more paranoid concerns. In the clinical domain it is consistent with observations of relative ignorance about technology in those presenting with ‘internet’ delusions.

However, while lack of familiarity and knowledge predict content to delusional ideation, other predictors of paranoia may well not apply, at least in the same way, to information technology. Freeman’s widely cited cognitive model of paranoia (Mordini, 2007) based on a review of available evidence, asserts that a set of internal (emotion/cognitive/anomalous experiences) and external (life event/trauma/drugs) factors interact together to lead to persecutory beliefs. These include the presence of hallucinatory experiences, perceptual anomalies, reasoning biases (need for closure, jumping to conclusions), and emotional processes (anxiety, depression, self-focus, interpersonal sensitivity). Although the present study did not directly assess what predicts cyber-paranoia from this list, it is likely to be a more specialized sub-set of these factors.

What the pattern of results also suggest is that there may be different drivers to cyber-fears, and to cyber-paranoia in particular, in different groups: lower technological awareness and use predict these in the general population, whereas for a highly IT literate group, trait paranoia exerts a stronger influence. Given the association with lower technological awareness, future research should assess whether accurate information provision and education about information technologies leads to a reduction of these fears.

There are several limitations to the study: it is based on a fairly technologically literate and relatively youthful sample – the pattern of results may well differ in other populations and these are deserving of study. There were insufficient respondents to examine the factorial validity of the measure and it may well be that some items more genuinely reflect paranoid concerns (intention of others to cause harm *and* heightened perception of threat). The sample is relatively small for testing the dimensional structure of a new scale and only EFA could be used. Further study in a second sample for testing the two-factor solution by confirmatory factor analysis is a necessary addition. Trait paranoia is a complex and multi-faceted construct and only a single rather general measure was used in the present study. More recent measures have stressed persecutory beliefs that may be very relevant to areas such as social networking in particular. Use of the scale in a social networking context would be of significant interest.

Our final point is a conceptual one, as it may be argued that a fear should only be labeled truly paranoid if it is clearly false (though this may often be difficult to reliably ascertain). Oftentimes, new developments in information and communication technology may be unusual in that the genuine scope for their use and misuse are still in the process of being fully explored both by their developers and users. Indeed, the boundaries between what is and is not possible with regards to technology are becoming increasingly blurred to the extent that even information experts debate them (Lewis and Fox, 2001; Ohm, 2008). It is difficult to be categorical about their potential for surveillance, malign use against the person or social control – factors that may make them potent breeding groups for conspiracy theories and widespread fears that may blend the ‘genuine’ and ‘false.’

## CONCLUSION

We present a new measure of cyber-fear/paranoia for general population use, which appears to be somewhat distinct from general trait paranoia. Factor analysis suggested the presence of two inter-correlated factors that we have termed cyber-fear and cyber-paranoia. In contrast to trait paranoia, cyber-fear/paranoia tended to increase with age and decrease with knowledge/use of technology. The distinctiveness of these fears and paranoias from general trait paranoia appears to mirror the clinical distinctiveness of ‘internet’ and other technology-fuelled delusions. Knowledge provision to increase technological proficiency and awareness may bring about a reduction in cyber-fear/paranoia.

## Conflict of Interest Statement

The authors declare that the research was conducted in the absence of any commercial or financial relationships that could be construed as a potential conflict of interest.
